# Endophyte-inoculated rhizomes of *Paris polyphylla* improve polyphyllin biosynthesis and yield: a transcriptomic analysis of the underlying mechanism

**DOI:** 10.3389/fmicb.2023.1261140

**Published:** 2023-10-30

**Authors:** Qing Zhang, Sheng Chang, Ying Yang, Congfang Xi, Yumei Dong, Lufeng Liu, Yunchao He, Yu Liu, Bo Cai, Tao Liu

**Affiliations:** ^1^National-Local Joint Engineering Research Center on Germplasm Innovation and Utilization of Chinese Medicinal Materials in Southwest China, College of Agriculture and Biotechnology, Yunnan Agricultural University, Kunming, China; ^2^Center of Yunnan Zhongyan Industry Co., Ltd., Kunming, China; ^3^Lushui City Katma Township People's Government Agricultural and Rural Integrated Service Center, Lushui, Yunnan, China; ^4^Shenzhen TCM Hospital, Shenzhen, China

**Keywords:** *Paris polyphylla*, endophyte, polyphyllin biosynthesis, transcriptome, beneficial interaction

## Abstract

**Introduction:**

Polyphyllin from *Paris polyphylla* var. *yunnanensis* exhibits anti-inflammatory, analgesic, antibacterial, and antiviral properties. However, the current production of polyphyllin can barely meet market demand. To improve the content of polyphyllin produced by *P. polyphylla*, two endophyte strains, *Bacillus cereus* LgD2 and *Fusarium oxysporum* TPB, were isolated from *Paris fargesii* Franch. and inoculated in the roots of *P. polyphylla*. Both symbiotic strains significantly promoted the accumulation of saponins in *P. polyphylla*.

**Methods:**

The content of polyphyllin in rhizomes of *P. polyphylla* treated with TPB with LgD2 strain was determined using High Performance Liquid Chromatography and the expressed genes were analyzed by RNA-seq. Gene Ontology and Kyoto Encyclopedia of Genes annotations were performed on the differentially expressed genes, a clustering tree of UDP-glycosyltransferase (UGT) and cytochrome P450 (CYP450) gene families was constructed, and UGT and CYP450 involved in the biosynthesis of polyphyllin were predicted using weighted correlation network analysis (WGCNA).

**Results:**

RNA-seq and qRT-PCR analyses showed that endophytic inoculation did not promote polyphyllin accumulation by enhancing the upstream terpene biosynthesis pathway, but probably by up-regulating the downstream CYP450 and UGT genes associated with polyphyllin biosynthesis. Genomes enrichment analyses of differentially expressed genes indicated that inoculation with LgD2 and TPB played a positive role in promoting the defense against pathogenic bacteria, enhancing the biosynthesis of carbohydrates, attenuating the process of nitrogen metabolism, and maintaining the equilibrium of the redox reaction homeostasis, potentially indirectly enhancing the polyphyllin yield of *P. polyphylla*. By combining differentially expressed genes screening, WGCNA, and phylogenetic tree analyses, 17 CYP450 and 2 UGT candidate genes involved in the biosynthesis of polyphyllin I, polyphyllin II, polyphyllin VII, polyphyllin D, and polyphyllin H were identified. These results suggest that endophytes probably effectively promote the accumulation of polyphyllin by regulating key downstream genes in biosynthetic pathways.

**Discussion:**

This study provides a new approach for investigating the regulatory mechanisms of endophytes that promote the production and accumulation of polyphyllin in *P. polyphylla*, providing a basis for further elucidating the mechanisms of plant-endophyte interactions.

## 1. Introduction

*Paris polyphylla* var. *yunnanensis* produces polyphyllin, which is an important ingredient used in traditional Chinese medicine, such as Gongxin, Yunnan Baiyao, Jinfu Kang Oral Liquid, and Lou Lian Capsules (Ren et al., 2020; Yan et al., 2021). Steroidal saponins, which mainly include isospirostanol-type saponins such as polyphyllin, diosgenin, and pennogenin, are important medicinal active ingredients derived from *P. polyphylla* (Thapa et al., 2022).

Polyphyllin I (PPI), polyphyllin II (PPII), polyphyllin VII (PPVII), polyphyllin H (PPH), and polyphyllin D (17-hydroxygracillin, PPD) are known to exert antitumor, anti-inflammatory, analgesic, antibacterial, antiviral, hemostatic, immune, and other therapeutic effects (Chen T. et al., 2019; Lin et al., 2021; Meng et al., 2021; Li Z. et al., 2022). For example, PPII has been reported to inhibit colorectal carcinogenesis by regulating mitochondrial fission and the activity of NF-κB pathways (Chen M. et al., 2019); PPVII exerts anti-inflammatory effects (Zhang et al., 2019), inhibits the proliferation of cell lines, and induces apoptosis and autophagy (Xiang et al., 2022); and PPH enhances blood clotting in the body and was also shown to treat acute myelogenous leukemia (Lu et al., 2018; Wen et al., 2019). Unfortunately, the slow growth and overexploitation of *P. polyphylla* have led to resource scarcity in Yunnan, resulting in domestic and international demand exceeding current supply (Negi et al., 2014). Even with widespread artificial cultivation, the amounts of active ingredients isolated from *P. polyphylla* cannot meet market demand; therefore, finding ways to increase the yield of polyphyllin in *P. polyphylla* appears to be a feasible method to counteract the imbalance in supply and demand.

Plant endophytes promote the growth and production of secondary metabolites of host plants, directly or indirectly, through various pathways (Santoyo et al., 2016; Worsley et al., 2020; Chen et al., 2021). They also produce bioactive compounds similar to the secondary metabolites of the host (Li et al., 2023). For example, the endophytic fungus *Pseudodidymocyrtis lobariellae* KL27, isolated from red buds, promotes the biosynthesis and accumulation of paclitaxel (Cao et al., 2022). Likewise, endophytes isolated from *Miquelia dentata Bedd*, produce camptothecin (Shweta et al., 2013).

An effective way for endophytes to promote the accumulation of polyphyllin in *P. polyphylla* is by enhancing the expression of genes in the polyphyllin biosynthetic pathway (Cao et al., 2022; Li Y. et al., 2022). Two pathways are involved in the biosynthesis of the terpene skeleton of polyphyllin, the cytoplasmic mevalonate (MVA) and plastidic 2-C-methyl-D-erythritol-methyl-D-erythritol-4-phosphate (MEP) pathways, among which the MVA pathway is dominant (Wang et al., 2015; Gao et al., 2020). Members of the cytochrome P450 (CYP450) and UDP-glycosyltransferase (UGT) gene superfamilies are required for the structural diversification of polyphyllin (Cheng et al., 2020), control of oxidation, and hydroxylation and glycosylation steps downstream of polyphyllin biosynthesis. Owing to the numerous members of these gene superfamilies, fully elucidating their roles in polyphyllin biosynthesis is difficult. However, RNA-seq technology can now be used to identify those gene members involved in polyphyllin biosynthesis (Cheng et al., 2020).

Despite the fact that endophytic fungi promote the growth, accumulation of secondary metabolites, and disease resistance of *P. polyphylla*, most studies have focused on growth promotion and disease resistance (Huang et al., 2009; Zhang et al., 2011; Tao et al., 2021). Few studies have investigated the effects of endophytes on the accumulation of polyphyllin in *P. polyphylla* and its mechanisms of action. *Bacillus cereus* LgD2 and *Fusarium oxysporum* TPB isolated from *Paris fargesii* Franch. play an important role in promoting the accumulation of polyphyllin (Yan et al., 2022). To further elucidate the biological mechanisms by which endophytes promote the accumulation of polyphyllin, the rhizomes of *P. polyphylla* inoculated with endophytes were subjected to RNA-seq analysis, and the candidate genes of the CYP450 and UGT superfamilies involved in the biosynthesis of polyphyllin were screened out. This study provides a theoretical basis for the regulatory role of endophytes in the accumulation of medicinal components in host plants and helps us better understand the role of CYP450s and UGTs in the postbiosynthetic modifications of polyphyllin. The findings provide a foundation for the development of biocides and *in vitro* polyphyllin biosynthesis.

## 2. Materials and methods

### 2.1. Activation and cultivation of endophytes

The endophytes *Bacillus cereus* LgD2 and *Fusarium oxysporum* TPB were isolated from *P. fargesii*, preserved in 50% glycerol, and stored at −80°C (Yan et al., 2022). Both LgD2 and TPB strains were removed and rapidly shaken at 37°C in a water bath for 10 s for activation.

The activated LgD2 bacterial solution was aspirated and inoculated in LB agar medium using the four-zone delineation method and incubated overnight at 37°C. A single colony was inoculated into 2 mL LB liquid medium (Solarbio Life Sciences, China) and incubated at 37°C for 5 h with shaking. Subsequently, 1 mL of this culture was inoculated into a triangular flask containing 500 mL liquid medium and shaken at 200 rpm at 37°C until the OD_600_ was 0.5–0.6. The culture was then centrifuged at 8.93 *g* for 5 min, and 500 mL of sterile water was added to resuspend the pellet and create a bacterial suspension (Costa Júnior et al., 2020; Liu et al., 2022).

An activated TPB mycelium was selected and inoculated into Potato Dextrose Agar medium (Solarbio Life Sciences, China) and cultured at 28°C for 5–7 days. The surface of the mycelium was rinsed with sterile water, and then filtered through four layers of sterile gauze to obtain a spore suspension (Andrade et al., 2018; Wang et al., 2021). The spore suspension was diluted in sterile water to 1 × 10^7^ cells/mL and kept for subsequent experiments.

### 2.2. Planting and endophyte inoculation of *Paris polyphylla*

On April 1, 2022, two-year-old *P. polyphylla* seedlings were purchased from Yunnan Suixitang Biotechnology Co. (China). They were planted in sterile plastic pots containing nutrient soil sterilized at 121°C for 1 h. Five plants were placed in each plastic pot and provided with 100 mL sterile water every 2–3 days. The pots were placed in the backyard greenhouse of Yunnan Agricultural University, 50% of the area of which was covered by a shade net, and its top was closed by a transparent plastic film. The strain inoculation treatments were carried out on May 1, 2022, with roots inoculated with bacterial or spore suspension every 15 dpi; five pots were inoculated for each treatment, with 50 mL of bacterial or spore suspension being poured into each pot separately. Sterile water (50 mL) was used as the control treatment. The rhizomes of *P. polyphylla* inoculated with endophyte strains three and six times (45 dpi and 90 dpi treatments) were collected on June 14, 2022 and July 29, 2022, respectively, cleaned, and immediately stored in liquid nitrogen.

### 2.3. RNA extraction

Three replicates of each of 45 dpi and 90 dpi rhizomes of *P. polyphylla* treated with LgD2, TPB, or sterile water (18 samples in total) were removed from liquid nitrogen and ground (0.1 g for each rhizome sample). RNA was extracted from the rhizomes of *P. polyphylla* using the HiPure HP Plant RNA Mini Kit (Guangzhou Magen Biotechnology, China) RNA integrity was assessed using 1% agarose gel electrophoresis and an Agilent 5,300 Bioanalyzer (Agilent, USA).

### 2.4. Library construction and sequencing

Briefly, 10 μg of total RNA of acceptable integrity, concentration, and purity was selected from each rhizome sample; 18 samples were used to construct libraries using the TruSeq Stranded mRNA LT Sample Prep Kit. Transcriptome sequencing was performed at Wuhan SeqHealth Technology Co., Ltd. (China), using an Illumina HiSeq 4,000 platform. The raw data obtained from MGISEQ-T7 sequencing were converted into sequence data using base calling (FASTQ format) to obtain the most original sequencing data file.

### 2.5. *De novo* transcriptome assembly and read notes

*De novo* transcriptome assembly and splice variant calling were performed using the Trinity software to obtain transcript sequences, with the longest transcripts as Unigenes. The SortMerna software was used to filter rRNA reads, which were error-corrected and deduplicated using the Rcorrector and fastUniq software. Data quality control was performed using fastp (version 0.23.0). Transcript splicing quality was evaluated using BUSCO (version 5.5.0).

### 2.6. Functional annotation and enrichment analysis

Protein sequences obtained from the Coding sequence (CDS) prediction by Unigene were annotated using the primary mission of Universal Protein Resource (UniPprot), Non-Redundant (NR), Pfam, evolutionary genealogy of genes: Non-supervised Orthologous Groups (eggNOG), Gene Ontology (GO),^1^ Kyoto Encyclopedia of Genes and Genomes (KEGG),^2^ and CAZYmes databases. Unigene sequences predicted without CDS were created in the NR and Rfam databases for annotation. Fragments per kilobase per million (FPKM) and read count values were calculated for each Unigene using Bowtie2 and eXpress. The absolute value of log2(FC) > 1 and value of *p* <0.05 were used as criteria to indicate that the gene was a differentially expressed Unigene. Hierarchical clustering analysis of differentially expressed genes (DEGs) was performed to determine the expression patterns of genes among different groups and samples. GO and KEGG pathway enrichment analyses were performed for DEGs using hypergeometric distribution-based R.

### 2.7. Construction of phylogenetic tree

The BLAST software and Pfam databases were used to identify transcripts belonging to CYP450 and UGTs. These transcripts were then translated into protein sequences using TBtools (version 1.120). The CYP450 protein sequences of *Arabidopsis thaliana* downloaded from TAIR,^3^ whereas the UGT sequences of *A. thaliana*, *Isatis tinctoria*, *Malus domestica*, *Carthamus tinctorius*, *Avena strigosa*, and *Arachis hypogaea* were downloaded from the NCBI database. A phylogenetic tree of CYP450 and UGT gene members was constructed using the maximum likelihood method (Chen et al., 2020). Phylogenetic topology was assessed using 1,000 bootstrap replicates.

### 2.8. Construction of gene coexpression networks

Gene co-expression networks were constructed using the weighted gene co-expression network (WGCNA) method in R software (version 3.2.2) (Langfelder and Horvath, 2008; Li et al., 2022). Genes with FPKM <1 in 90% of samples were filtered out, and the expression matrices of the top 8,000 genes were selected as input files in WGCNA for the identification of gene modules with strong co-expression. By calculating the correlation coefficients and clusters of the levels of expression of each sample, samples with low correlation or those that could not be clustered on the tree diagram were removed. The WGCNA network was constructed and modules were detected based on the phenotypic traits of polyphyllin. Candidate CYP450 and UGT genes were selected from the important templates most relevant to the phenotypic data, based on edge and KME values assigned to the assumed hub genes of the polyphyllin biosynthesis network. Final visualization was performed using Cytoscape (version 3.10.0) (Doncheva et al., 2019).

### 2.9. Quantitative real-time fluorescence PCR (qRT-PCR)

Total RNA of acceptable purity and integrity was used for the synthesis of first-strand cDNA using the All-in-One RT 5× MasterMix (ABM, Canada) alongside the AccuRT Genomic DNA Removal Kit. The EvaGreen 2X qPCR MasterMix kit (ABM, Canada) was used to validate the levels of expression of five genes related to the steroid saponin biosynthesis pathway. Primers were designed using Primer3Plus^4^ (Supplementary Table S1). The reaction system (20 μL) consisted of 10 μL of 2× SYBR Premix Ex Taq™, 2 μL cDNA template, 0.5 μL of upstream and downstream primers, and 7 μL of ddH_2_O. PCR conditions were as follows: initial denaturation at 95°C for 2 min, followed by 40 cycles of denaturation at 95°C for 10 s, annealing at 60°C for 15 s, and extension at 72°C for 15 s. Four biological replicates were used for each treatment. The relative gene expression was analyzed using the 2^-ΔΔCq^ method and β-actin was used as the internal reference gene (Bustin et al., 2005; Nolan et al., 2006).

### 2.10. PPI, PPII, PPVII, PPH, and PPD content determination

According to the standard for the determination of the medicinal content of *P. polyphylla* in “Chinese Pharmacopoeia 2020 Edition,” high-performance liquid chromatography (General 0512) was used to determine the content of PPI, PPII, PPVII, PPH, and PPD in *P. polyphylla*. Briefly, rhizome samples of *P. polyphylla* treated with TPB and LgD2 for 90 dpi were dried at 45°C to a constant weight, and then ground into powder using a Chinese herbal medicine pulverizer. Next, 0.5 g of powder (passed through sieve No. 3) was obtained, precisely weighed, and placed in a 50 mL volumetric flask. Then, 20 mL methanol was added to the flask, immersed for 30 min, ultrasonicated for 30 min for extraction, and cooled at 25°C. Additional methanol was used to make up for the loss of weight, and the mixture was shaken well, centrifuged at 8.93 *g* for 5 min, and then filtered through a 0.45 μm micropore filter membrane. The content of PPI, PPII, PPVII, PPH, and PPD in the solution was determined by high performance-liquid chromatography (HPLC) using a C18 column (Zorbax ODS 4.6 × 250 mm, 5 μm; Agilent Technologies, USA) at a detection wavelength of 203 nm, column temperature of 30°C, and flow rate of 1.0 mL/min. PPI, PPII, PPVII, PPH, and PPD controls were purchased from Sichuan Vicki Biotechnology Co. Ltd. (China). Appropriate amounts of PPI, PPII, PPVII, PPH, and PPD controls were obtained, precisely weighed, and mixed with methanol to obtain solutions containing 0.4 mg of each per 1 mL of methanol. For analysis, 20 μL of each of the control and test solutions were precisely aspirated, using octadecylsilane-bonded silica gel as filler and acetonitrile (solvent A) and water (solvent B) as the moving phase in a linear gradient: 0 min: 30% A; 40 min: 60% A; 41 min: 100% A; 44 min: 80% A (Yang et al., 2013; Yan et al., 2022).

### 2.11. Data analysis

IBM SPSS Statistics (version 25) was used to calculate the mean and standard deviation. An independent samples *t*-test was used to statistically evaluate the differences between the two treatments. *Asterisks indicate significant differences from the control group (*t*-test; ∗ *p* < 0.05; ∗∗ *p* < 0.01). The “heatmap” package in R (version 4.2.2) was used for clustering heatmap plots, whereas the “ggplot2” package was used for visualization.

## 3. Results

### 3.1. TPB and LgD2 inoculation promoted the accumulation of polyphyllin in *Paris polyphylla*

To investigate the potential effect of endophytes on polyphyllin biosynthesis in *P. polyphylla*, the content of polyphyllin in *P. polyphylla* was determined before and after treatment with TPB and LgD2. Inoculation with TPB and LgD2 led to the increased accumulation of polyphyllin in *P. polyphylla* (Supplementary Table S2). The contents of PPVII, PPD, PPH, and PPI were significantly increased by 42.77, 26.95, 21.96, and 15.27%, respectively, in the TPB treatment group. Likewise, the contents of PPVII, PPD, and PPH were significantly increased by 24, 69.38, and 19.06%, respectively, whereas the PPII content was significantly decreased by 45.97% in the LgD2 treatment group. These results indicated that inoculation with TPB or LgD2 significantly induced the biosynthesis and accumulation of polyphyllin in *P. polyphylla*.

### 3.2. RNA-seq assembly and annotation

To identify the intrinsic mechanism by which the TPB and LgD2 strains promote polyphyllin biosynthesis, total RNA isolated from the rhizomes of *P. polyphylla* was sequenced under different treatments using the Illumina HiSeq 4,000 platform. Eighteen sequencing libraries constructed from three biological replicates each of 45 and 90 dpi TPB-, LgD2-treated, and control rhizome samples yielded a total of 861,041,222 clean data reads. The Q30 of each sequencing data set was >95%, and 788,438,886 dedup reads were retained after UID deduplication. Accordingly, 1,329,726 contigs were obtained by assembling the dedup reads using Trinity, with an N50 of 638 bp, average length of 537.49 bp, longest read length of 16,964 bp, and average GC content of 45.11%. BUSCO analysis suggested 81% coverage and completeness for transcriptome assembly (Table 1). Then, 1,262,695 (94.96%) of these Unigenes were annotated using the KEGG, UniProt, NR, Pfam, Rfam, eggnog, and GO databases (Supplementary Table S3). The transcriptome sequence data were stored in the NCBI Short Read Archive (SRA) under the BioProject registration number PRJNA935848.

**Table 1:** Summary of sequences obtained by RNA-seq analysis of *Paris polyphylla*.

Names	Number
Total of raw reads	976,402,382
Clean reads	861,041,222
Dedup reads	562,331,692
Total base	714,719,250
GC percentage (%)	45.11
Number of contigs	1,329,726
Maximum length of contigs (bp)	16,964
Minimum length of contigs (bp)	180
Average length of contigs (bp)	537.49
N50 of contigs (bp)	638
Number of Unigenes	1,038,548
Total BUSCO groups searched	2,326
Complete BUSCO (%)	81.5
Missing BUSCO (%)	12.4

### 3.3. Functional annotation and enrichment analysis of DEGs

**Figure 1:**
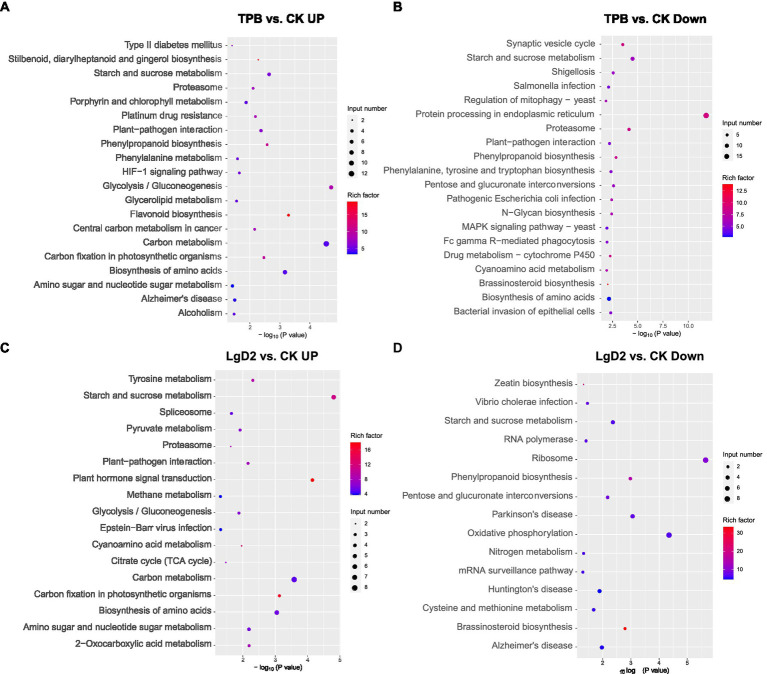
Bubble plots of KEGG functional enrichment for *Paris polyphylla* under TPB treatment, **(A)** upregulated vs. **(B)** downregulated; and *P. polyphylla* under LgD2 treatment **(C)** upregulated vs. **(D)** downregulated compared with the control. The size and color of circles in the graph indicates the number of genes and enrichment factor, respectively; a more intense color indicates more enrichment.

DEGs were screened from plants inoculated with TPB and LgD2 strains and functional annotation was performed. Gene expression was significantly different between plants under treatment with different strains and inoculation times (Supplementary Figure S1). GO annotation of DEGs revealed that TPB treatment significantly upregulated phosphorus metabolism, protein modification, oxidation–reduction, and phosphotransferase, methyltransferase, acyltransferase, and metal ion transmembrane transporter activities, whereas nitrogen metabolism, RNA metabolism, metal ion binding, and cation binding were significantly downregulated. Additionally, LgD2 treatment upregulated oxidation–reduction processes, responses to abiotic stimuli, cellular redox homeostasis, carbohydrate and ATP biosynthesis, phosphotransferase, and UDP-glycosyltransferase activities, whereas carbohydrate metabolism, purine nucleotide metabolism, and metal and oxygen binding were downregulated (Supplementary Figure S2). KEGG pathway analysis showed that TPB treatment upregulated DEGs mainly enriched in the pathways of carbon fixation, glycolysis/gluconeogenesis, and plant-pathogen interaction in photosynthetic organisms. Conversely, downregulated DEGs were mainly enriched in the pathways of bacterial invasion of epithelial cells, pathogenic *E. coli* and *Salmonella* infections, and shigellosis. LgD2 treatment also upregulated carbon metabolism, the tricarboxylic acid (TCA) cycle, plant interactions with pathogens, *Vibrio cholerae* infection, nitrogen metabolism, oxidative phosphorylation, and other pathways (Figure 1). These analyses revealed that TPB and LgD2 treatment promoted carbohydrate accumulation and redox homeostasis, increased the levels of phosphorylation, glycosylation, and acylation, and reduced the binding capacity to metal ions and cations. In particular, plant-pathogen interactions were significantly upregulated, whereas the ability of some pathogenic bacteria for infection and bacterial invasion of epithelial cells were decreased, suggesting that endophytic inoculation may promote the defense response of *P. polyphylla* against some bacteria and pathogens.

### 3.4. Inoculation with TPB and LgD2 alters the terpene backbone and steroid biosynthetic pathway

**Figure 2:**
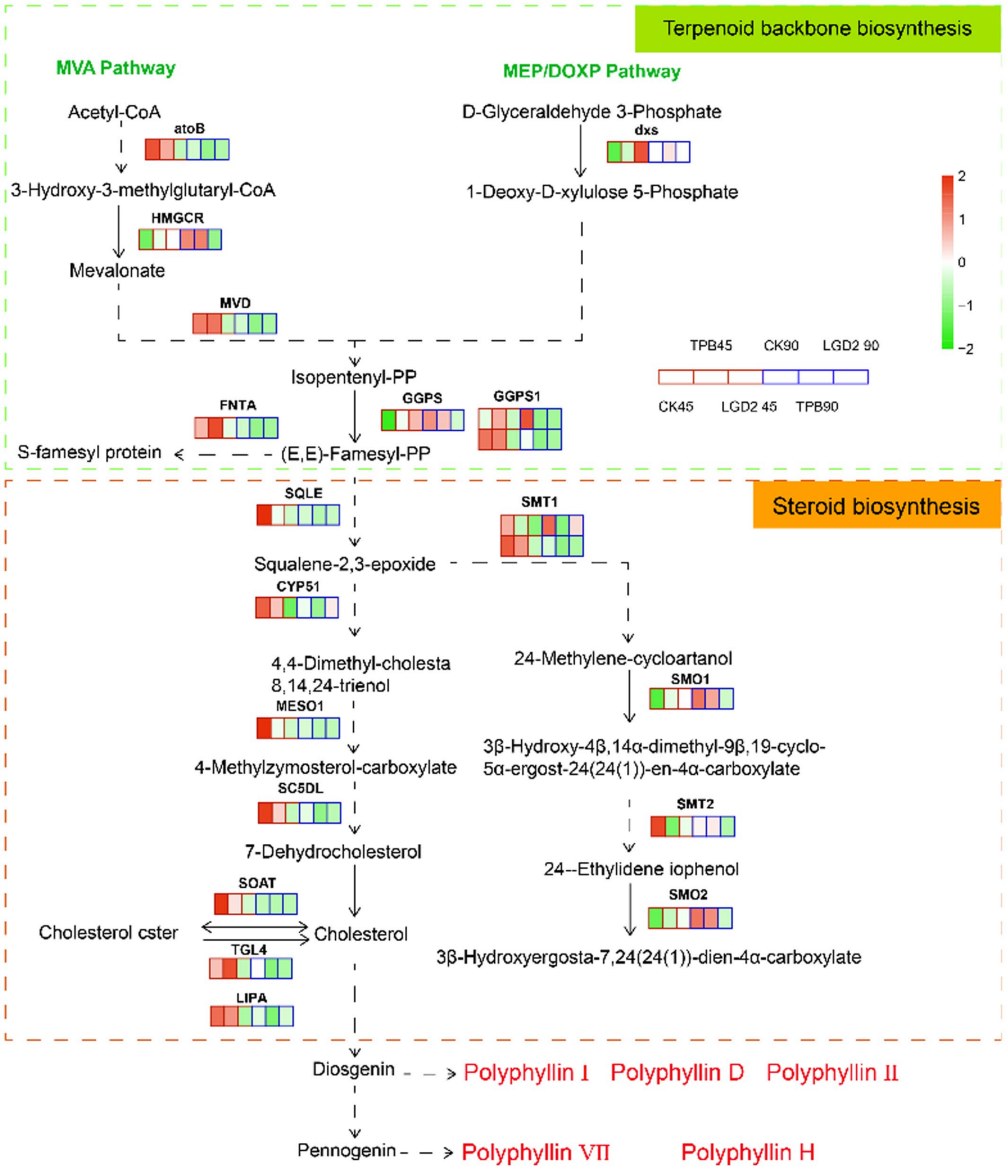
Terpene skeleton and steroid biosynthesis pathways with expression map. The heatmap highlights the expression patterns of these genes under treatment with different strains and at different times; RPKM values were used to normalize the corresponding color coding.

The terpene backbone and steroid biosynthesis pathways are located upstream of polyphyllin biosynthesis. According to the annotation using the KEGG database, 722 Unigenes were identified as involved in “steroid biosynthesis” (ko00100), encoding 31 enzymes, and while 823 Unigenes were identified as related to “terpenoid skeleton biosynthesis” (ko00900), encoding 36 enzymes. Twenty Unigenes were highly homologous to the functional signature and DEGs under different strain treatments. More specifically, eight DEGs were present in the terpene skeleton biosynthesis pathway, whereas 12 DEGs were present in the steroid biosynthesis pathway (Figure 2).

Unexpectedly, the remaining genes, except farnesyltransferase type-1 subunit alpha (FNTA), 1-deoxy-D-xylulose-5-phosphate synthase (dxs), and TAG lipase acyltransferase (TGL4), were downregulated under strain treatment compared with those in the control. An increased number of genes and differential genes were annotated to the MVA pathway, corroborating that the MVA pathway is the main pathway for polyphyllin biosynthesis (Liu et al., 2016; Upadhyay et al., 2018). These genes were downregulated at 90 dpi, except for hydroxymethylglutaryl-CoA reductase (HMGCR), geranylgeranyl diphosphate synthase (GGPS), plant 4,4-dimethylsterol C-4alpha-methyl-monooxygenase (SMO1), and plant 4alpha-monomethylsterol monooxygenase (SMO2). Previous studies have shown that PPI, PPII, and PPD are diosgenins, whereas PPVII and PPPH are pennogenins, all of which are synthesized from cholesterol (Deng et al., 1999; Li et al., 2001). Therefore, cholesterol accumulation is key to the biosynthesis and accumulation of polyphyllin. Strain treatment did not significantly promote cholesterol biosynthesis; it had the opposite effect, showing a decreasing tendency in the levels of cholesterol over time.

To further validate the transcriptomic results, the expression of five key genes involved in polyphyllin biosynthesis was investigated. The qRT-PCR analysis revealed that the expression of four genes involved in cholesterol biosynthesis, 3-hydroxy-3-methylglutaryl coenzyme A reductase (HMGR), squalene synthase (SQS), cycloartenol synthase (CAS), and oxidosqualene cyclase (SE), was downregulated in *P. polyphylla* under TPB and LgD2 treatment. The only exceptions were the *CAS* gene at 45 dpi rhizomes under TPB treatment and the *SE* gene at 90 dpi rhizomes treated with LgD2 (Supplementary Figure S3). Overall, the genes upstream of polyphyllin biosynthesis mainly showed a trend of downregulated expression under different strain inoculations and time treatments, which was basically consistent with our RNA sequencing analysis, indicating reliable the RNA-seq results. CYP90B27 is responsible for C-22 hydroxylation in the steroidal alkaloid biosynthesis pathway, a downstream pathway in polyphyllin biosynthesis (Yin et al., 2018). The expression of the *CYP90B27* gene was upregulated in *P. polyphylla* treated with either strain at 90 dpi, which may be important for the accumulation of polyphyllin.

### 3.5. Phylogenetic analysis of the CYP and UGT gene families in *Paris polyphylla*

**Figure 3:**
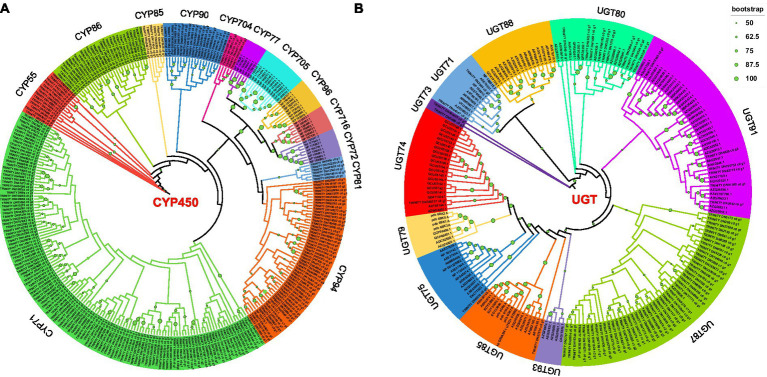
Phylogenetic tree of **(A)** CYP450s and **(B)** UGTs. Predicted amino acid sequences of CYP450s and UGTs in *P. polyphylla* were aligned with selected CYP450s and UGTs from other plant species using MUSCLE. The evolutionary history was inferred using the maximum-likelihood method. The bootstrap consensus tree inferred from 1,000 replicates represents the evolutionary history of the taxa analyzed.

In this study, Pfam annotation and the BLAST algorithm were used to identify 257 CYP450s and 71 UGTs. Forty-five CYP450s were downloaded from the *Arabidopsis thaliana* database and used to construct a phylogenetic tree; in total, 302 CYP450s were characterized into 13 subfamilies (Figure 3A). Through phylogenetic analysis, the 257 CYP450s of *P. polyphylla* were assigned to the CYP71, CYP94, CYP81, CYP704, CYP90, CYP85, CYP86, and CYP55 families. Among them, CYP71 and CYP94 had the largest number of CYPs, followed by CYP86, CYP90, and CYP55, whereas CYP704 had the lowest number of CYP450s. Additionally, 132 UGTs of *A. thaliana*, *Isatis tinctoria*, *Malus domestica*, *Carthamus tinctorius*, *Avena strigosa*, and *Arachis hypogaea* were downloaded and used to construct a phylogenetic tree. In total, 203 UGTs were classified into 11 subfamilies (Figure 3B). The 71 UGTs of *P. polyphylla* belonged to the UGT74, UGT75, UGT85, UGT87, UGT91, UGT80, UGT71, and UGT73 families. Among them, UGT87 and UGT80 had the highest number of UGT genes, followed by UGT91, UGT74, and UGT75, whereas UGT85 had the lowest number of genes.

**Figure 4:**
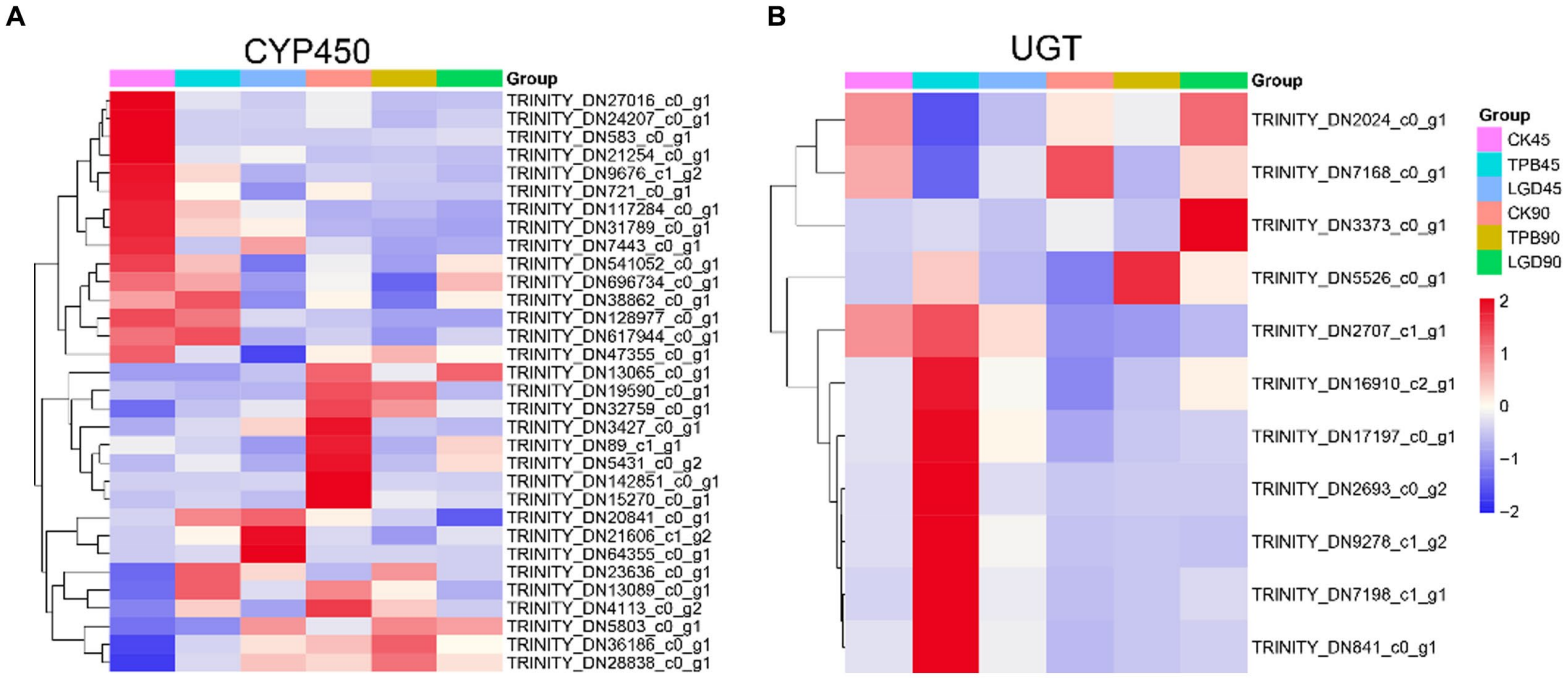
Heatmap of the expression of CYP450 **(A)** and UGT **(B)** genes in *P. polyphylla* under treatment with different strains and at different times. RPKM values were used to normalize the corresponding color coding.

In total, 257 CYP450s and 71 UGTs genes were screened for differential expression and 32 CYP450s and 11 UGTs were differentially expressed between the control and TPB and LgD2 treatments. These differentially expressed CYP450s were grouped into three categories, with the most common being CYP71, followed by CYP86 and CYP94 (Figure 4A). Interestingly, genes in the first category were highly expressed in the control at 45 dpi, those in the second category were highly expressed in the control at 90 dpi, whereas those in the third category were upregulated at both 45 and 90 dpi under TPB and LgD2 treatments. Similarly, UTGs were grouped into two categories, with ten genes belonging to UGT87, whereas one was assigned to UGT80 (Figure 4B). The first class of UGTs was upregulated at 90 dpi, whereas the second class was highly expressed at 45 dpi in plants under TPB treatment. Differentially expressed CYP450s were both up- and down-regulated, whereas differentially expressed UGTs were mostly upregulated at different times and under different treatments.

Annotation of these CYP450 and UGT genes to the Uniprot, NR, eggNog, GO, KEGG, and CAZYmes databases (Supplementary Table S4) revealed that the functions of these differentially expressed UGTs were mainly related to the formation of glycosidic bonds (GTs), with GT1 accounting for the major part. GO annotation showed that among them, TRINITY_DN2024_c0_g1 and TRINITY_DN2707_c1_g1 belonged to the metabolic pathway of secondary metabolite biosynthesis, functioning in the transfer of hexosyl groups. Moreover, eggNOG database annotations revealed that TRINITY_DN2707_c1_g1 has a flavonol 3-O-glucosyltransferase activity. TRINITY_DN2024_c0_g1 was annotated as scopoletin glucosyltransferase-like protein in the NR database. Annotation of TRINITY_DN541052_c0_g1 and TRINITY_DN117284_c0_g1, which are differentially expressed CYP450 genes, suggested that they function as GT31 and GT1, respectively, in the CAZYmes database. NR annotations identified TRINITY_DN541052_c0_g1 as a lanosterol 14-alpha-demethylase and TRINITY_DN117284_c0_g1 as an isoflavone 2′-hydroxylase-like enzyme.

### 3.6. Prediction of functional genes by gene coexpression analysis and polyphyllin content

**Figure 5:**
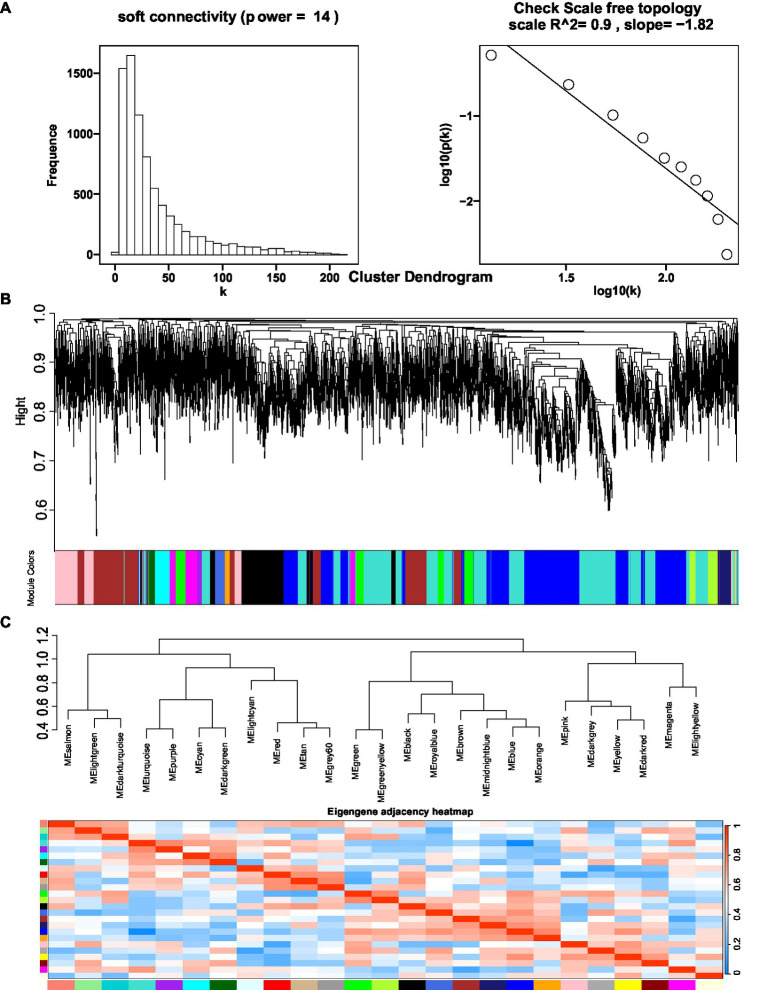
Optimal soft threshold map **(A)**, clustered dendrogram **(B)**, and eigengene adjacency heatmap **(C)** for WGCNA analysis. The hierarchical gene clustering method was used to organize the clustering tree of coexpression network modules based on the 1-tom matrix. Each module uses a different color.

**Figure 6:**
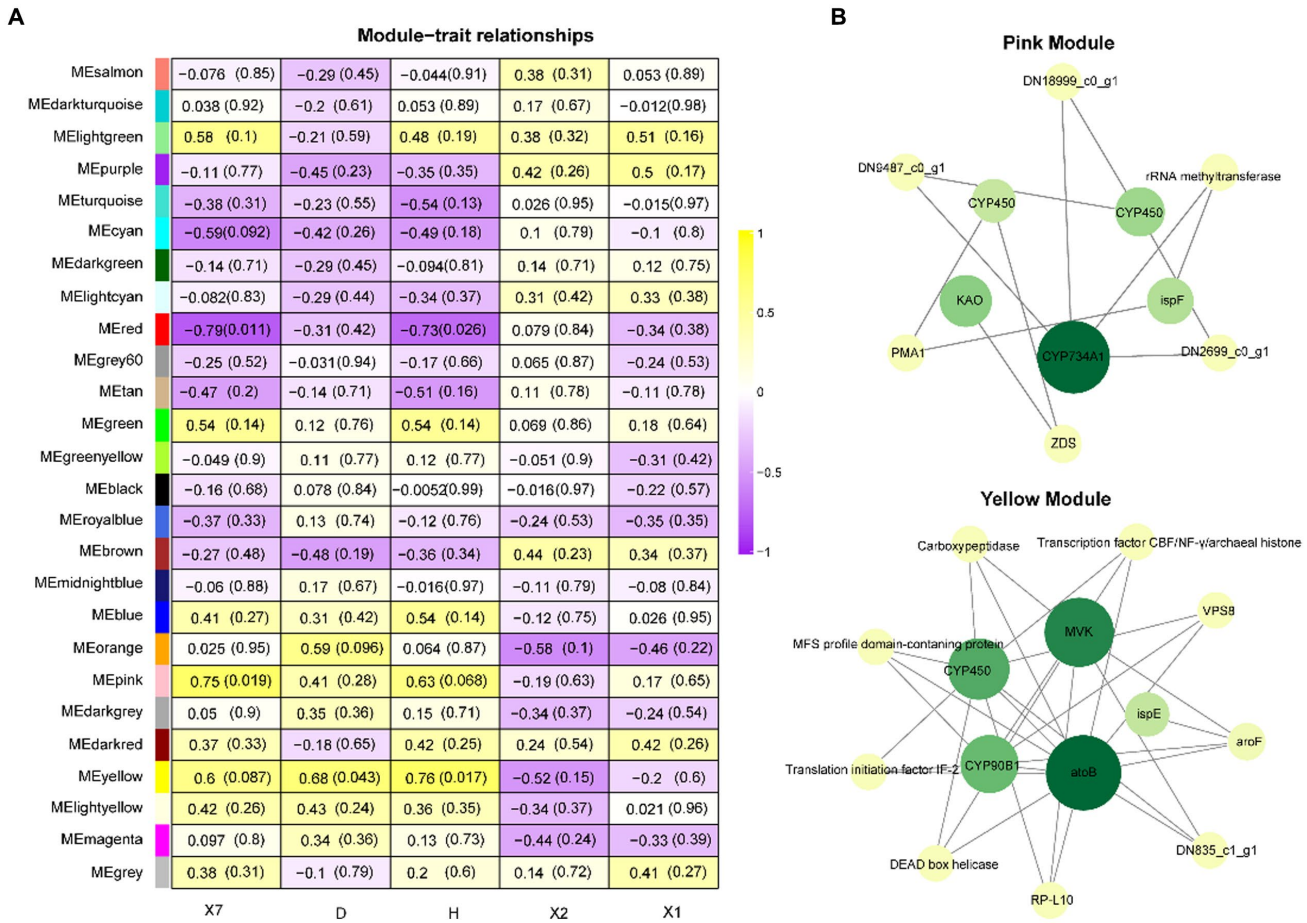
WGCNA analysis. **(A)** Heatmap of WGCNA coexpression module-trait association. The rows and columns correspond to the module of the trait genes and metabolites, respectively. Each cell contains the correlation and value of p of the gene in the module with the corresponding metabolite. **(B)** Hub gene correlation network diagram.

The coexpression network was constructed by inputting transcript data from *P. polyphylla* under different treatments and choosing a soft threshold, *β* = 14, at which *R*^2^ = 0.9, indicating the successful generation of the model (Figure 5A). Unigenes with similar expression patterns were clustered in the same branch, with different colors indicating different coexpression modules in the branch (Figure 5B). A threshold of 30 was chosen for each module, and the 8,000 Unigenes were divided with the highest correlation into 26 modules (Figure 5C). Then, the genes in the steroid and terpene backbone biosynthesis pathway were identified by selecting the expression modules that were significantly associated with polyphyllin content, and the correlation coefficients between the biosynthesis of polyphyllin and gene modules were calculated (Figure 6A). The closer the correlation coefficient was to one, the more similar the expression or distribution patterns of the metabolites and genes in the modules of *P. polyphylla* inoculated with different strains. Among them, PPVII and PPH were most correlated with the pink module, with correlation values of 0.75 and 0.76, respectively; PPD was most correlated with the yellow module (0.68); PPII was most correlated with the brown module (0.44); and PPI was most correlated with the light green module (0.51), indicating that the genes in the pink, yellow, brown, and light green modules were highly correlated and might be involved in polyphyllin biosynthesis. In total, 1880 genes were co-clustered with the expression modules in the four modules.

Of the 1880 genes, were selected and screened those involved in the terpene skeleton and steroid biosynthesis pathways to identify genes shared in the CYP450 and UGT phylogenetic trees. Using the four prediction modules, six genes in the terpene skeleton biosynthesis pathway, six genes in the steroid biosynthesis pathway, 17 CYP450s, and 2 UGTs were identified. The clustering heatmap shows the expression patterns of these genes under different treatments (Supplementary Figure S4). The expression of these genes was upregulated under TPB and LgD2 treatment, especially that of CYP90B1, which may play a key role in promoting polyphyllin biosynthesis.

To explore the function of these CYP450 and UGT genes, these candidate genes were annotated into the Uniprot, NR, eggNog, GO, KEGG, and CAZYmes databases (Supplementary Table S5). Annotation in the CAZYmes database revealed that TRINITY_DN2002_c0_g1 and TRINITY_DN19349_c0_g1 were both GT1, whereas NR database annotation resulted in their identification as UDP-glycosyltransferase 92A1-like and UDP-glycosyltransferase 89B1, respectively. TRINITY_DN7787_c1_g1 was annotated to the diterpenoid biosynthesis pathway in the KEGG database and to GT31 in the CAZYmes database, and Uniprot and NR annotations revealed that this gene encodes an Ent-kaurenoic acid oxidase.

To further investigate the roles of CYP450s and UGTs in polyphyllin-related modules, the top genes with the highest weights in the pink and yellow modules were selected for network correlation analysis. CYP450, KAO, CYP734A1, and ispE play key roles in the pink module, whereas CYP450, acetyl-CoA C-acetyltransferase (atoB), 4-diphosphocytidyl-2-C-methyl-D-erythritol kinase (ispE), CYP90B1, and mevalonate kinase (MVK) play key roles in the yellow module (Figure 6B).

## 4. Discussion

Endophytes, such as bacteria and fungi, are microorganisms that interact with host plants. Complex microbial communities are beneficial for host nutrient supply, plant growth hormones, promotion of metabolite accumulation, and control of soil-borne and systemic pathogens (Deng and Cao, 2017; Dudeja et al., 2021; Pandey et al., 2022). Hence, endophytes have potential applications in the pharmaceutical, agricultural, and food industries because of their ability to enhance the levels of biologically active secondary metabolites in the host and produce metabolites similar to those of the host (El-Sayed et al., 2020). Endophytes can form long-term, stable, and mutually beneficial symbiotic relationships with medicinal plants, influencing the secondary metabolism of the plant and its therapeutic properties (Maggini et al., 2017). Endophytes of the genera *Fusarium*, *Nodulisporium*, *Brevundimonas*, and *Bacillus*, isolated from *Panax notoginseng*, can produce major ginsenosides (Luo et al., 2013).

Polyphyllin is the main medicinal active ingredient in the perennial medicinal plants of *P. polyphyllin* listed in the Chinese Pharmacopeia. However, owing to its slow growth, *P. polyphyllin* has a low polyphyllin content, whereas *P. fargesii*, a closely related species of the same genus, has a high polyphyllin content. Therefore, it is of great significance to isolate and screen *P. fargesii* strains to identify the specific factors that promote the accumulation of polyphyllin content in *P. polyphyllin*, and increase the accumulation of its medicinal active ingredients. The endophytes LgD2 and TPB isolated from *P. fargesii* have the potential to promote the biosynthesis of polyphyllin in *P. polyphyllin*. Although *F. oxysporum* is a pathogen of most plants and can cause wilt, root-, and foot-rot in many plant species, as a root endophyte, it can also inhibit diseases caused by vascular pathogens and pathogenic *F. oxysporum* strains owing to a long-term interactive relationship with the host plant, further protecting the host plant against root pathogens from other plant endophytes (de Lamo and Takken, 2020).

After inoculating *P. polyphylla* with TPB and LgD2 by root irrigation, the accumulation of polyphyllin in the rhizomes was significantly increased at 90 dpi (Supplementary Table S2). In addition, TPB treatment had the strongest positive effect on pennogenins (PPVII and PPH), whereas LgD2 treatment had the strongest effect on diosgenins (PPI, PPII, and PPD). The effects of these two strains on the biosynthetic pathway of polyphyllin in *P. polyphylla* differed. Moreover, the increase in the content of PPD and decrease in that of PPII under LgD2 treatment were simultaneous, indicating that the change in the content of PPD was likely attributed to the conversion of the original PPII biosynthetic pathway.

To reveal the intrinsic mechanism of endophytic inoculation on polyphyllin biosynthesis in *P. polyphylla*, 45 dpi and 90 dpi treated *P. polyphylla* were selected for RNA-seq and qRT-PCR analysis (Yan et al., 2022). A total of 1,329,726 Unigenes were annotated from 18 sequencing libraries. Annotation and enrichment analyses of the identified DEGs showed that the activities of auxin biosynthesis, protein modification, methylation, acylation, and phosphorylation were increased in plants under TPB treatment. Methylation, UDP-glycosylation, and phosphorylation were upregulated in *P. polyphylla* under LgD2 treatment (Figure 1). In plant environments, microorganisms can interact with host plants and synthesize specific metabolites. Epigenetic modifications are key regulators of plant chromatin structure and gene expression, controlling plant growth and development, as well as responses to various environmental stresses (Wang et al., 2020). Various coenzymes catalyze redox, group transfer, and allosteric reactions. In addition, methylation, acylation, and glycosylation of secondary metabolites catalyzed by O-methyltransferases allow the modification of secondary metabolites (Cheynier et al., 2013). Endophytic bacteria upregulate protein modification, methylation, acylation, and glycosylation in plant hosts, suggesting that endophytes enhance plant resistance and may play an important role in the modification of secondary metabolites.

Both treatments enhanced the redox reactions, TCA cycle, and carbohydrate biosynthesis in *P. polyphylla* (Figure 1). Redox reactions in plants play an important role in photosynthesis. The nitrogen-fixing reaction in the photosystem is the most important part of redox reactions in plant cells and plays a crucial role in photosynthesis. The TCA cycle is the ultimate common oxidation pathway for carbohydrates, fats, and amino acids. It is the most important metabolic pathway for supplying energy to the body (Akram, 2014). As plant growth occurs through carbon fixation (Ducat and Silver, 2012), the observed increase in carbon fixation capacity implied that both strains have a facilitative effect on photosynthesis. Carbohydrates are the basis for plant biomass and yield (Chandel, 2021), and the accumulation of photosynthetic products is the result of increased photosynthetic capacity, indicating that the TPB and LgD2 strains had positive effects on the growth of *P. polyphylla*. In addition, increased plant-pathogen interactions decreased bacterial invasion in epithelial cells, and infection by pathogenic *E. coli*, *Salmonella*, and *Shigella,* suggesting that endophytic bacteria also play a role in the disease resistance and defense response of the host plant.

qRT-PCR analysis revealed that the four genes involved in cholesterol biosynthesis, HMGR, SQS, CAS, and SE, were downregulated, whereas CYP90B27, which is involved in C-22 hydroxylation, was upregulated in plants after strain treatment. RNA-seq results also revealed that the expression of Unigenes, which was annotated to the terpene skeleton and steroid biosynthesis pathways, was not increased by strain treatment, whereas an increase was mainly observed in CYP450 and UGT genes (Figures 2, 4). Hence, the increase in the content of polyphyllin in rhizomes of *P. polyphyllin* treated with TPB and LgD2 was mainly attributed to the modification of cholesterol, such as glycosylation and hydroxylation.

Many members of the CYP450 and UGT superfamilies are involved in the biosynthesis of steroids and polyphyllin and play key roles in the conversions of PPI, PPII, PPVII, PPD, and PPH. CYP716A53v2 is a protopanaxadiol 6-hydroxylase that catalyzes the production of PPT from PPD in ginseng (Park et al., 2016). CYP72A397 is an oleanolic acid 23-hydroxylase involved in the production of oleanolic acid in *Kalopanax septemlobus* (Han et al., 2018). CYP90B1 is a steroidal C-22 hydroxylase that catalyzes the early C-22 hydroxylation of various C sterols including C27, C28, and C29 (Fujita et al., 2006; Cheng et al., 2020). UGTs, which are the largest family of glycosyltransferases in higher plants that modify secondary metabolites and hormones, mediate the transfer of glycosyl residues from activated nucleotide sugars to glycosidic elements. This family of genes plays an important role in the broad diversity of species-specific plant secondary metabolites (Ross et al., 2001; Nishimura et al., 2010).

Using WGCNA, the genes of the steroidal saponin and terpene backbone biosynthesis pathways that were most significantly affected under strain treatment were selected. Combined with the phylogenetic tree, the CYP450 and UGT genes in the coexpression module, which are likely to be responsible for the differences in polyphyllin content; however, these results need further validation. To find the key CYP450 and UGT candidate genes, The screened genes were functionally annotated. Annotation to the CAZYmes database revealed that these genes mainly function as GTs (glycosidic bond formation), of which GT1 accounted for the major portion, followed by GT31. The GT1 family consists of a variety of enzymes that catalyze the monosaccharide addition of different compounds. The 11 differentially expressed screened UGTs were identified to function as GT1, of which, except for TRINITY_DN7168_c0_g1, were more highly expressed under strain treatment. The glycosylation modification of these UGTs may play a key role in the accumulation of PPI, PPII, PPVII, PPD, and PPH. Moreover, the action of TRINITY_DN7168_c0_g1 resulted in a decrease in the accumulation of PPI, PPII, PPVII, PPD, and PPH, and may be a paralogous branch for the synthesis of poliphyllin.

Under the induction of endophytes, host plants may improve the polyphyllin yield by regulating genes related to the polyphyllin biosynthesis pathway. In the present study, the transcriptomes of *B. cereus* LgD2 and *F. oxysporum* TPB-inoculated rhizomes of *P. polyphylla* were sequenced. Enrichment analysis of DEGs revealed that endophyte inoculation promoted the chemical modification of downstream metabolites and had positive effects on the growth and disease resistance of *P. polyphylla*. Phylogenetic trees and WGCNA revealed CYP450s and UGTs that may be involved in the biosynthesis of PPI, PPII, PPVII, PPD, and PPH; however, their specific functions and roles must be further validated.

## 5. Conclusion

This study showed that the endophytes, *B. cereus* (LgD2) and *F. oxysporum* (TPB), promote the accumulation of polyphyllin in *P. polyphylla* and facilitate beneficial plant-endophyte interactions. In addition, endophyte-plant associations increased the production of polyphyllin by enhancing the expression of CYP450s and UGTs. This study revealed the effects of *B. cereus* and *F. Oxysporum* on plant metabolism and related regulatory processes and screened for CYPs and UGTs that affect polyphyllin biosynthesis. These findings provide a theoretical basis for improving the medicinal value of Chinese herbal medicines and help to further elucidate plant-endophyte interactions and biosynthetic pathways of polyphyllin.

## Data availability statement

The data presented in this study are deposited in the NCBI Sequence Read Archive (SRA) under accession number PRJNA935848 (https://www.ncbi.nlm.nih.gov/sra/?term=PRJNA935848).

## Author contributions

QZ: Writing – original draft. SC: Writing – review & editing, Formal analysis, Validation. YY: Writing – review & editing, Formal analysis, Validation. CX: Data curation, Investigation, Writing – review & editing. YD: Writing – review & editing. LL: Validation, Writing – review & editing. YH: Writing – review & editing. YL: Formal analysis, Resources, Writing – review & editing. BC: Writing – review & editing, Methodology, Resources. TL: Conceptualization, Methodology, Funding acquisition, Writing – original draft.

## Footnotes

^1^http://www.geneontology.org

^2^http://www.genome.jp/kegg/

^3^https://www.arabidopsis.org/

^4^https://www.primer3plus.com/
