# Supplementary material for: Endophyte-inoculated rhizomes of Paris polyphylla improve polyphyllin biosynthesis and yield: a transcriptomic analysis of the underlying mechanism
Source: Front Microbiol. 2023 Oct 30;14:1261140. doi: 10.3389/fmicb.2023.1261140 (PMC10643526; doi:10.3389/fmicb.2023.1261140)
Supplement: Supplementary file 1 [file Presentation_1.pdf]

Supplementary Material

1 Supplementary Figures and Tables

1.1 Supplementary Tables

Supplementary Table 1: Gene and primer sequences.

Gene	Primer	Sequence
β-Actin	Forward	CAGCAGATGTGGATCTCAAAGG
	Reverse	GCGAACAATCATAACCAAGCA
HMGR	Forward	TTCCCCGATATGTACGTGATCAG
	Reverse	CTTGAGAACCTTCTTCACCACCT
CYP90B27	Forward	GCCATCGCCCTAGCTATCTATTT
	Reverse	GGGTGAACTCCATTTGCTTGTAG
SQS	Forward	GGAGAACTCTACAAATGCCTTGC
	Reverse	GATTGCCATGATCTGGGGAATTG
SE	Forward	GGACAGGCCTGCTTCGATTA
	Reverse	TCAGTTGGCGGGAGTTCTTC
CAS	Forward	CTCTTGGGCTGTTTGCTTCAC
	Reverse	CCAACCACCAGAAGGCAACT

Supplementary Table 2: TPB and LgD2 strains inoculation treatment versus control for polyphyllin content. Values are means ± SD, significance tests were performed using independent samples t-test, * and ** indicate significant ($P < 0.05$) and highly significant ($P < 0.01$) differences, respectively.

content(mg/g)	CK	TPB	LgD2
**PPVII**	6.37±0.25	9.10±0.64*	7.90±0.54**
**PPD**	0.92±0.03	1.17±0.07**	1.56±0.13**
**PPH**	7.40±0.37	9.03±0.81*	8.81±0.45*
**PPII**	10.45±0.68	10.09±1.11	5.64±0.89**
**PPI**	6.00±0.29	6.91±0.11**	4.63±0.82

Supplementary Table 3: Unigenes annotations of the predicted CDS.

DataBase	Number	Percent (%)
Uniprot	586342	44.09
NR	592366	44.55
Pfam	664465	49.97
Rfam	1123118	84.46
eggNog	494701	37.20
GO	435503	32.75
KEGG	179978	13.54
**Total**	1262695	94.96

1.2 Supplementary Figures

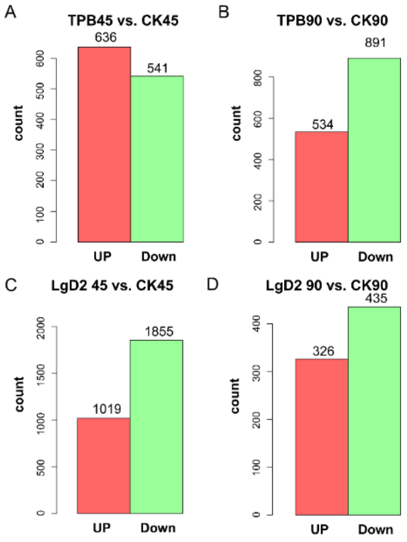

Supplementary Figure 1. DEGs correlation scatter plots. A: CK 45dpi vs. TPB 45dpi; B CK 90dpi vs. TPB: 90dpi; C CK 45dpi vs. LgD2 45dpi; D CK 90dpi vs. LgD2 90dpi.

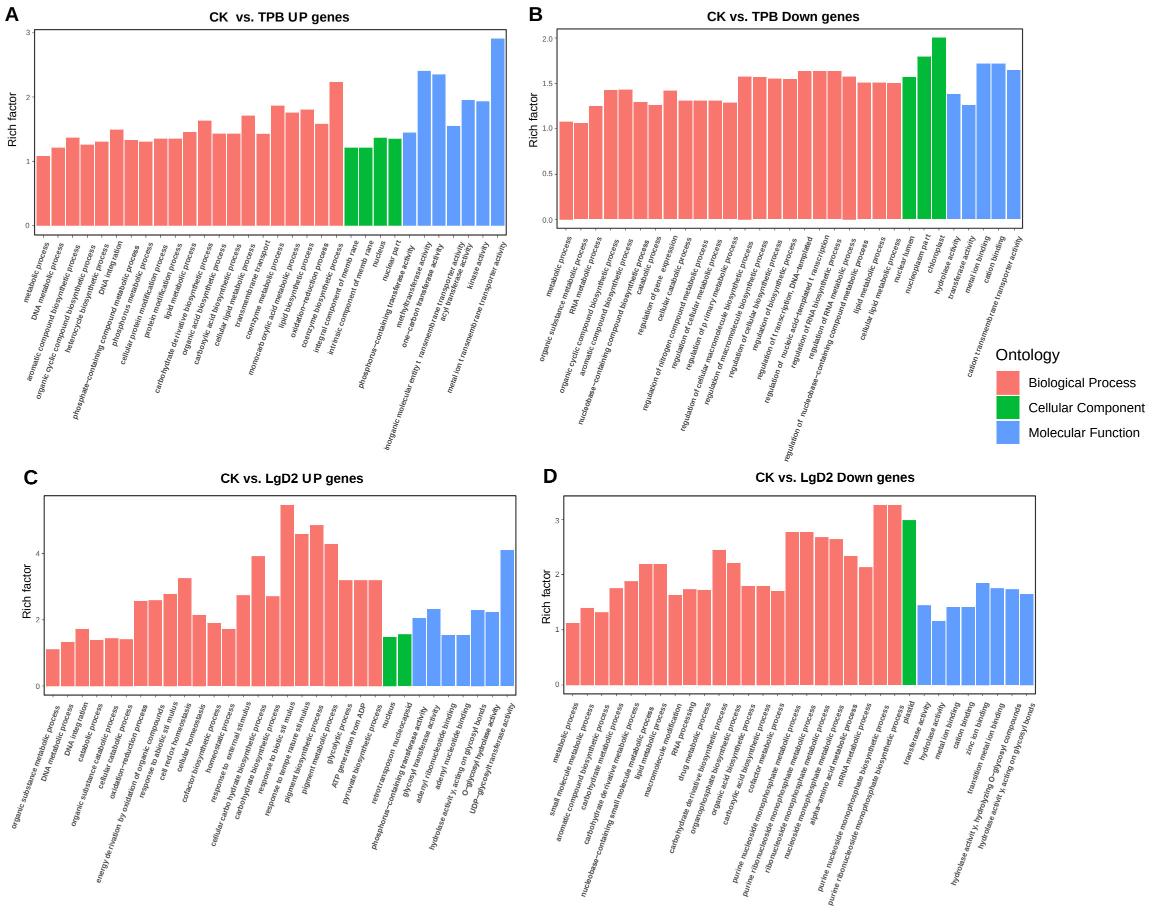

Supplementary Figure 2. GO enrichment terms of DEGs. A: CK vs. TPB UP genes; B CK vs. TPB Down genes; C CK vs. LgD2 UP genes; D CK vs. LgD2 Down genes.

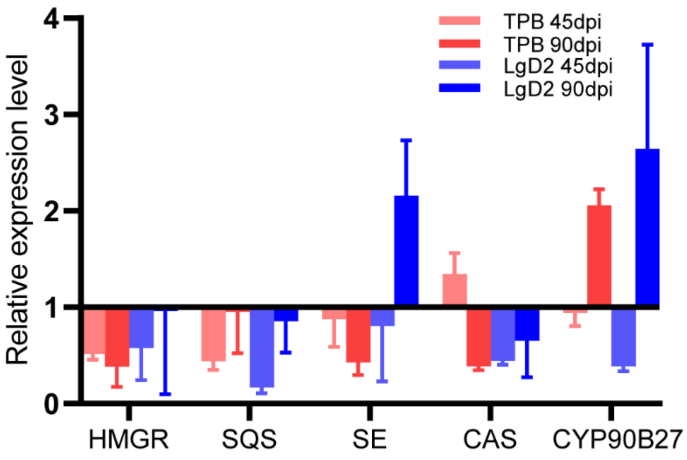

Supplementary Figure 3. Relative expression levels of genes related to terpenoid skeleton synthesis. Compared with CK, relative expression levels below 1 in the graph are down-regulated expression and above 1 is up-regulated expression. The relative expression levels of genes were assessed using the $2^{-\Delta\Delta Ct}$ method.

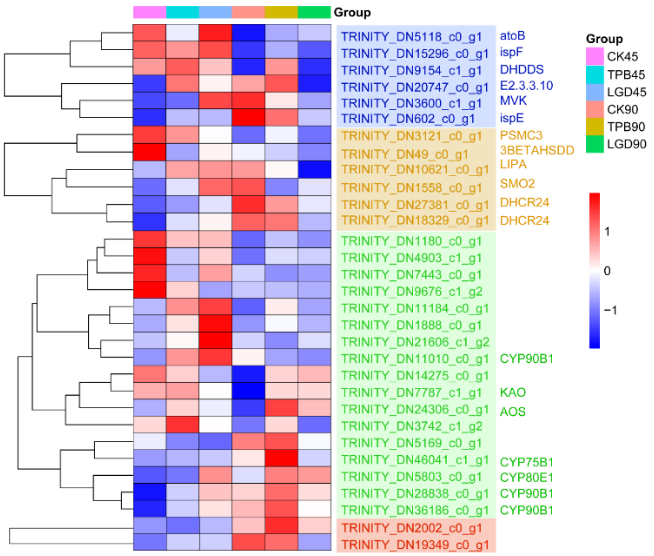

Supplementary Figure 4. Heat map of gene clustering of the biosynthetic pathway genes in the four modules of WGCNA for polyphyllin. Blue, yellow, green and red in the figure represent the gene ID and KEGG annotation names of terpene backbone biosynthesis, steroid biosynthesis, CYP450 and UGT, respectively.
